# Effectiveness of a two-stage posterior-anterior–posterior surgery using subcutaneously preserved autologous bone grafts for adult spinal deformity: a retrospective observational study

**DOI:** 10.1186/s13018-024-04572-8

**Published:** 2024-01-27

**Authors:** Atsushi Kimura, Yasuyuki Shiraishi, Hideaki Sawamura, Hirokazu Inoue, Katsushi Takeshita

**Affiliations:** Department of Orthopaedic Surgery, Jichi Medical University, 3311-1 Yakushiji, Shimotsuke, Tochigi 329-0498 Japan

**Keywords:** Adult spinal deformity, Two-stage surgery, Perioperative complication, Autologous bone graft, Subcutaneous preservation

## Abstract

**Background:**

To achieve optimal correction of rigid kyphoscoliosis, we developed a novel two-stage posterior-anterior–posterior (PAP) surgery using subcutaneously preserved autologous bone grafts. This study aimed to investigate the effectiveness of two-stage PAP surgery versus single-stage anterior–posterior (AP) surgery.

**Methods:**

This was a retrospective analysis of patients undergoing combined anterior–posterior long-level fusion for adult spinal deformity (ASD) with a minimum 2-year follow-up. The indications for two-stage PAP surgery were rigid thoracolumbar deformity associated with hypertrophic facet arthritis and/or a large pelvic incidence–lumbar lordosis mismatch of > 25°. In the first stage of PAP surgery, pedicle screw insertion and multilevel Ponte osteotomies were performed. The resected local bone was embedded under sutured fascia. One week later, the embedded bone was retrieved in the right lateral position and used as an autograft for lateral lumbar interbody fusion. Final deformity correction was performed in the prone position.

**Results:**

From January 2018 to April 2021, 12 and 16 patients with ASD underwent two-stage PAP surgery (PAP group) and single-stage AP surgery (AP group), respectively. Although PAP surgery was associated with a significantly longer operation time, the total blood loss volume was significantly less in the PAP group than the AP group. Compared with the AP group, the PAP group showed significantly larger postoperative changes in radiological parameters in the sagittal and coronal planes. The overall complication rate did not differ significantly between the two groups.

**Conclusion:**

Two-stage PAP surgery provided effective correction of rigid kyphoscoliosis without increasing blood loss and postoperative complication rates.

## Background

Adult spinal deformity (ASD) is most commonly diagnosed in patients older than 60 years and has multiple etiologies, including progressive degeneration of the discs, facet joints, and paraspinal muscles. ASD ultimately leads to global misalignment and lumbar canal stenosis, which typically cause severe back pain, radicular pain, gait impairment, and reflux esophagitis [1]. As ASD has become increasingly recognized in developed countries, the demand for ASD surgery has grown rapidly, especially among patients older than 65 years [2].

A key factor in successful ASD surgery is to obtain an optimal lumbar lordosis (LL) that is harmonized with the pelvic incidence (PI) [3]. Specifically, a postoperative PI–LL mismatch of < 10° results in balanced sagittal alignment and improved health-related quality of life [3, 4]. Successful realignment of ASD often requires long-level fusion surgery from the thoracic spine to the sacrum combined with various correction techniques, including multilevel facet osteotomies, three-column osteotomy, and combined anterior–posterior (AP) surgery [5, 6]. These techniques enable powerful correction of thoracolumbar deformity; however, they also involve a prolonged operative time, increased blood loss, and increased risk of perioperative complications.

Several recent studies have demonstrated that patients who undergo combined AP surgery using multilevel lateral lumbar interbody fusion (LLIF) have better functional outcomes and lower complication rates than patients treated with an all-posterior approach [7, 8]. While combined AP surgery typically starts with multilevel LLIF, severe thoracolumbar deformity is commonly associated with hypertrophic facet arthritis, which may interfere with the opening of intervertebral space. We hypothesized that completing the posterior release prior to LLIF using staged posterior-anterior–posterior (PAP) procedures improves the efficacy of LLIF and reduces the risk of endplate injury. Therefore, we developed a novel two-stage PAP surgery for ASD using subcutaneously preserved local bone grafts. This study aimed to investigate the effectiveness of two-stage PAP surgery versus single-stage AP surgery for ASD.

## Methods

### Patients

After approval was obtained from the Institutional Review Board of our hospital, patients who underwent surgical treatment for ASD between January 2018 and April 2021 were enrolled in a prospective database. We then retrospectively analyzed the data to investigate the effectiveness of two-stage PAP surgery compared with single-stage AP surgery. The inclusion criteria were: (1) age older than 50 years; (2) long spinal fusion (from the thoracic spine to the sacrum with S2 alar-iliac (AI) screws) using multilevel LLIF with a minimum of follow-up of 2 years; and (3) at least one of the following radiological spinopelvic parameters: coronal Cobb angle > 20°; sagittal vertical axis (SVA) > 50 mm; pelvic tilt (PT) > 20°; and T1 pelvic angle (TPA) > 20°. The exclusion criteria were: (1) three-column osteotomy; (2) iatrogenic spinal deformity; (3) history of adolescent idiopathic scoliosis; (4) pyogenic spondylitis; and (5) comorbidities that impair physical functions (e.g., brain infarction, severe rheumatoid arthritis, and Parkinson disease).

### Two-stage PAP surgery

**Fig. 1:**
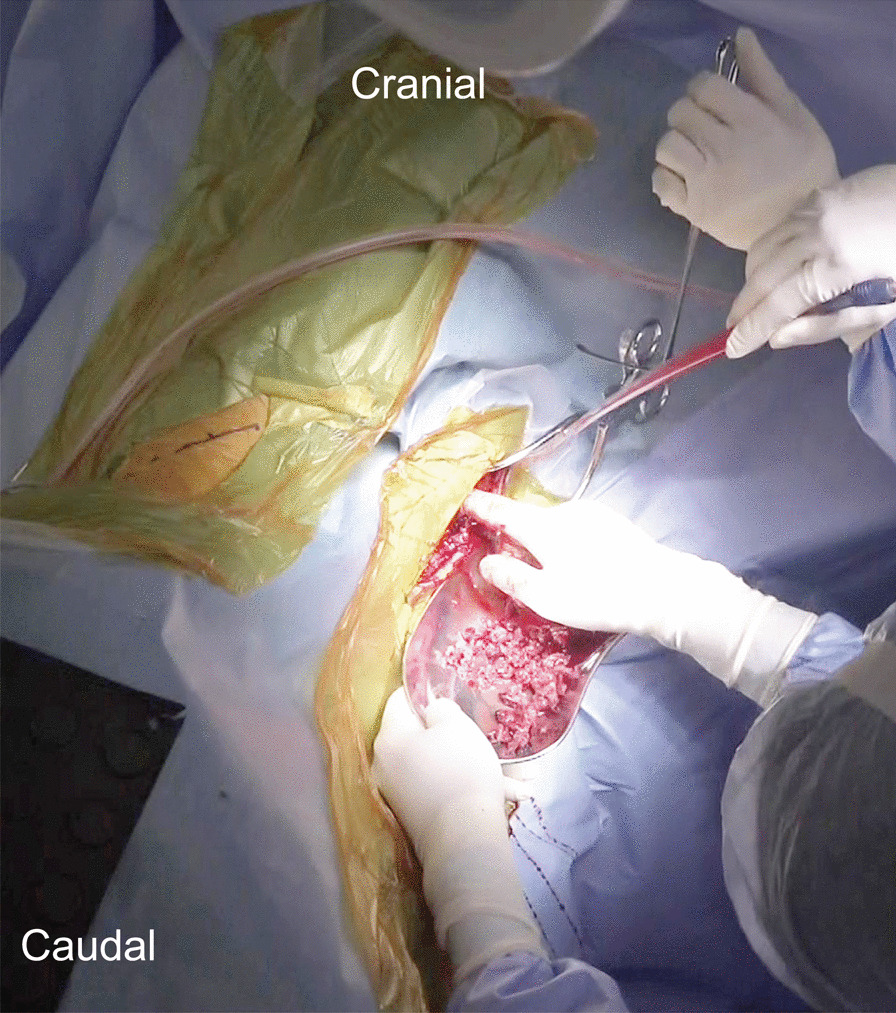
Retrieval of local bone grafts. To minimize the number of postural changes, the subcutaneously preserved local bone graft was retrieved through partial suture removal of the posterior wound in the right lateral decubitus position

The indications for two-stage PAP surgery were rigid thoracolumbar deformity associated with hypertrophic facet arthritis and/or a PI–LL mismatch of > 25°. Two-stage PAP surgery started with the placement of pedicle screws and S2 AI screws through a midline skin incision with the patient in a prone position. Next, posterior release was achieved by multilevel Ponte osteotomies. Autologous local bone fragments harvested from the osteotomies were placed under sutured fascia at the thoracic and upper lumbar levels. During the interval between the staged surgeries, patients were encouraged to get out of bed and exercise to avoid complications associated with prolonged bedrest. The degree of back pain differed substantially between individuals, so the level of physical activity was set within the limits permitted by their back pain. The second-stage surgery was performed 7 days after the first surgery. To minimize the number of postural changes, the subcutaneously preserved local bone graft was retrieved through partial suture removal of the posterior wound in the right lateral decubitus position (Fig. 1). Then, poly-ether-ether-ketone (PEEK) LLIF cages filled with local bone were inserted into lumbar intervertebral spaces via the standard oblique pre-psoas retroperitoneal approach. Next, the patient was moved to the prone position, and L5/S1 posterior lumbar interbody fusion was performed using a lordotic cage loaded with local bone. The thoracolumbar deformity was corrected using rod-cantilever and/or rod-derotation techniques. The volume of preserved local bone graft was usually sufficient to load multiple LLIF cages, and the residual bone graft was used for posterolateral spinal fusion.

### Single-stage AP surgery

Patients who did not have the indications for two-stage PAP surgery received single-stage AP surgery. Single-stage surgery started with LLIF in the right lateral decubitus position. PEEK cages loaded with iliac crest bone graft (ICBG) were inserted into lumbar intervertebral disc spaces. If the ICBG volume was not enough to fill the PEEK cages, synthetic bone material (Mastergraft, Medtronic Sofamor Danek, Memphis, TN) was used. Next, the patient was moved to the prone position, and pedicle screw insertion and L5/S1 posterior lumbar interbody fusion were performed. Following multilevel Ponte osteotomies, the thoracolumbar deformity was corrected using rod-cantilever and/or rod-derotation techniques.

### Data collection

We collected data regarding patient demographics, surgical data, radiological parameters, and patient-reported outcomes. Surgical data included surgical strategy (two-stage PAP or single-stage AP), American Society of Anesthesiologists physical status (ASA-PS), levels of the upper instrumented vertebra (UIV) and lower instrumented vertebra, operative time, estimated blood loss (EBL), and hospital stay.

Full-length free-standing posteroanterior and lateral spine radiographs were obtained at specified time intervals (preoperatively and 4-weeks, 1-year, and 2-years postoperatively). Spinopelvic parameters, including thoracic kyphosis, LL, SVA, PI, PT, PI–LL, TPA, Cobb angle, and coronal balance distance (CBD) were measured. The CBD was defined as the horizontal distance between the C7 plumb line and the central sacral vertical line. The sagittal and coronal lumbar segmental Cobb angles before and after surgery were measured using computed tomography multiplanar reconstruction (CT-MPR), as described previously [9].

Complications were classified as perioperative or delayed, and as minor or major. Perioperative complications were those occurring within 6 weeks postoperatively, while delayed complications were those occurring between 6 weeks postoperatively and final follow-up. A complication was classified as major if it substantially prolonged hospitalization, involved an invasive intervention, required reoperation, or had prolonged or permanent morbidity, as defined previously [10]. The proximal junctional kyphosis (PJK) angle was defined as the sagittal angle subtended by the inferior endplate of the UIV and the superior endplate two levels above the UIV. PJK was defined by two criteria: (1) PJK angle ≥ 10° and (2) PJK angle 10° greater than the preoperative measurement [11]. Patient-reported outcomes were assessed using the Scoliosis Research Society–22 (SRS-22) questionnaire preoperatively and 2 years postoperatively.

### Statistical analysis

Between-group comparisons were performed using unpaired t-tests for means, Pearson’s Chi-squared tests for proportions, and Wilcoxon rank-sum tests for medians. Statistical analyses were performed using GraphPad Prism 7.0 (GraphPad Prism Software Inc., San Diego, CA) and JMP version 14 (SAS Institute, Inc., Cary, NC). For all statistical analyses, the level of significance was set at P < 0.05.

## Results

### Patient demographics

A total of 52 consecutive patients underwent corrective fusion surgery for ASD during the study period. Twenty-four patients were excluded because of short fusion levels (typically from L2 to the sacrum; *N* = 6), three-column osteotomy (*N* = 5), a history of adolescent idiopathic scoliosis (*N* = 4), iatrogenic spinal deformity (*N* = 3), a history of pyogenic spondylitis (*N* = 1), neurological comorbidities (*N* = 3), and loss to 2-year follow-up (*N* = 2). The final study cohort comprised 28 patients, including 12 who underwent two-stage PAP surgery (PAP group) and 16 who underwent single-stage AP surgery (AP group) (Table 1). Although age and ASA-PS were comparable in the two groups, the PAP group had a significantly lower height and weight than the AP group.

**Table 1:** Patient demographics

Variable	Two-stage PAP surgery (*N* = 12)	Single-stage AP surgery (*N* = 16)	*P* value*
Age	68.4 ± 4.2	66.4 ± 8.8	0.478
Sex (male/female)	1/11	1/15	0.832
Height (cm)	146.7 ± 6.8	154.6 ± 6.2	0.004
Weight (kg)	47.7 ± 8.4	57.7 ± 9.6	0.008
BMI (kg/m^2^)	22.0 ± 2.2	24.2 ± 3.7	0.088
ASA-PS			0.315
Class I	2 (17)	1 (7)	
Class II	9 (75)	15 (93)	
Class III	1 (8)	0 (0)	

Data are shown as number (%) or mean ± SD. PAP, posterior-anterior–posterior; AP, anterior–posterior; BMI, body mass index; ASA-PS, American Society of Anesthesiologists physical status. *P values were calculated using the unpaired t-test for means and the Chi-squared test for proportions

### Surgical data

The total operation time was significantly longer in the PAP group than the AP group (Table 2). In contrast, the total EBL was significantly lesser in the PAP group than in the AP group. The hospital stay did not significantly differ between the groups.

**Table 2:** Surgical data

Variable	Two-stage PAP surgery (*N* = 12)	Single-stage AP surgery (*N* = 16)	P value*
UIV level			0.090
T4	2 (17)	0	
T9	10 (83)	16 (100)	
UIV instrumentation			0.090
Hook	10 (83)	16 (100)	
Screw	2 (17)	0	
LIV instrumentation			1.000
S2 alar-iliac screw	12 (100)	16 (100)	
LLIF level			0.241
L1/2-L4/5	11 (92)	14 (87)	
L2/3–L4/5	0 (0)	2 (13)	
T12/L1–L3/4	1 (8)	0 (0)	
Ponte osteotomy level			0.241
L1/2-L4/5	11 (92)	14 (87)	
L2/3–L4/5	0 (0)	2 (13)	
T12/L1–L4/5	1 (8)	0 (0)	
Operation time (min)			
First stage	223.3 ± 47.8	449.1 ± 56.3	
Second stage	279.3 ± 47.5	N/A	
Total time	491.3 ± 42.0	449.1 ± 56.3	0.039
EBL (ml)			
First stage	706.7 ± 346.5	1681.1 ± 779.4	
Second stage	435.0 ± 195.3	N/A	
Total EBL	1141.7 ± 390.7	1681.1 ± 779.4	0.038
Hospital stay (days)	33.9 ± 6.7	31.7 ± 11.5	0.555

Data are shown as number (%) or mean ± standard deviation. PAP, posterior-anterior–posterior; AP, anterior–posterior; UIV, upper instrumented vertebra; LIV, lower instrumented vertebra; LLIF, lateral lumbar interbody fusion; EBL, estimated blood loss. *P values were calculated using the unpaired t-test for means and the Chi-squared test for proportions

The complications in the two groups are shown in Table 3. Perioperative major complications included deep wound infection that required reoperation in the AP group, and segmental motor weakness due to radiculopathy in both groups; the two patients with motor weakness experienced spontaneous recovery within 6 months postoperatively. Rod breakage was the most common major delayed complication in both groups. One patient in the AP group had incisional hernia at the site of iliac bone harvest. The overall complication rates did not differ significantly between the two groups.

**Table 3:** Complications in the two-stage PAP and single-stage AP groups

Operative stage	Severity	Category	Two-stage PAP surgery (*N* = 12)	Single-stage AP surgery (*N* = 16)	*P* value*
Perioperative (≤ 6 weeks)	Major	Deep wound infection	0 (0)	1 (6)	0.378
		Motor weakness	1 (8)	1 (6)	0.832
	Minor	Delirium	1 (8)	1 (6)	0.832
		Urinary infection	0 (0)	1 (6)	0.378
		Asymptomatic DVT	1 (8)	0 (0)	0.240
		Endplate injury	0 (0)	3 (19)	0.112
Delayed (> 6 weeks)	Major	Rod breakage	3 (25)	4 (25)	1.000
	Minor	PJK	2 (17)	3 (19)	0.887
		Incisional hernia	0 (0)	1 (6)	0.378
Any complication			6 (50)	9 (56)	0.743

Data are shown as number (%). PAP, posterior-anterior–posterior; AP, anterior–posterior; DVT, deep vein thrombosis; PJK, proximal junctional kyphosis. *P values were calculated using the Chi-squared test

### Radiographic data

**Fig. 2:**
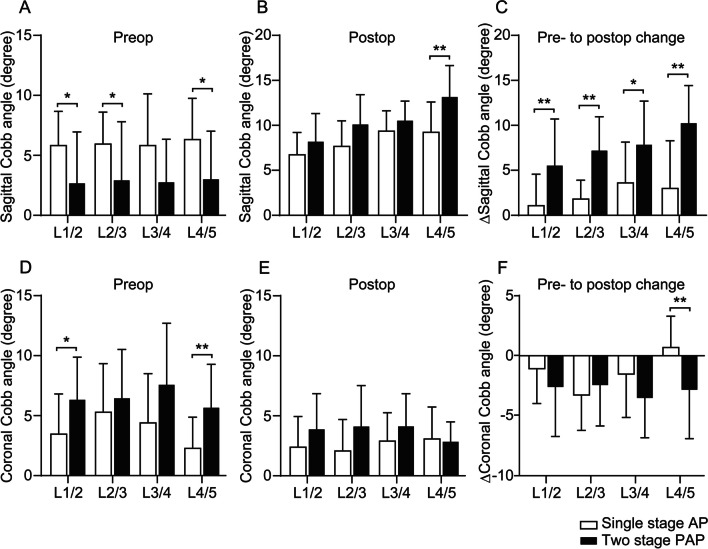
Segmental sagittal (**A**–**C**) and coronal (**D**–**F**) Cobb angles measured using CT-MPR. Despite the significantly smaller preoperative sagittal Cobb angles in the PAP group compared with the AP group (**A**), postoperative sagittal Cobb angle at the level of L4/5 was significantly larger in the PAP group than in the AP group (**B**). The postoperative changes in the sagittal segmental Cobb angles were significantly larger in the two-stage PAP group than in the single-stage AP group at all intervertebral levels (**C**). The change in the coronal segmental Cobb angle at the level of L4/5 was also significantly larger in the PAP group than in the AP group (**F**). *P < 0.05, **P < 0.01

The radiological parameters in the two groups are summarized in Table 4. The PAP group had a significantly larger LL, PT, PI–LL, Cobb angle, and CBD than the AP group at baseline. Despite the more severe preoperative deformity in the PAP group, the postoperative PI–LL was significantly smaller in the PAP group than the AP group, and the other radiological parameters became comparable between the two groups postoperatively. Compared with the AP group, the PAP group showed significantly larger postoperative changes in radiological parameters, including LL, PT, PI–LL, TPA, SVA, Cobb angle, and CBD. The sagittal and coronal lumbar segmental Cobb angles measured using CT-MPR are summarized in Fig. 2. Despite the significantly smaller preoperative sagittal Cobb angles in the PAP group than the AP group, the PAP group had a significantly larger postoperative sagittal Cobb angle at the L4/5 level than the AP group. Furthermore, the postoperative changes in the sagittal segmental Cobb angles were significantly larger in the two-stage PAP group than the single-stage AP group at all lumbar segmental levels. The change in the coronal segmental Cobb angle at the L4/5 level was also significantly larger in the PAP group than the AP group.

**Table 4:** Radiological parameters

Parameter	Two-stage PAP surgery (*N* = 12)	Single-stage AP surgery (*N* = 16)	*P* value*
Preoperative			
TK (°)	15.3 ± 10.0	17.6 ± 12.3	0.612
LL (°)	1.0 ± 12.6	16.4 ± 14.5	**0.007**
PT (°)	38.9 ± 10.2	32.4 ± 6.5	**0.048**
PI (°)	49.3 ± 8.1	51.1 ± 7.8	0.574
PI–LL (°)	44.8 ± 13.2	33.3 ± 15.7	**0.049**
TPA (°)	40.5 ± 10.9	32.6 ± 9.5	0.051
SVA (mm)	117.3 ± 17.0	89.1 ± 14.7	0.223
Cobb angle (°)	40.9 ± 15.4	23.4 ± 15.9	**0.007**
CBD (mm)	47.8 ± 35.7	22.8 ± 16.6	**0.020**
Postoperative			
TK (°)	35.2 ± 7.1	37.1 ± 8.0	0.508
LL (°)	42.3 ± 5.5	39.9 ± 8.0	0.382
PT (°)	21.9 ± 6.1	25.3 ± 4.9	0.114
PI (°)	49.1 ± 7.4	51.6 ± 7.3	0.373
PI–LL (°)	6.8 ± 5.7	11.7 ± 6.3	0.043
TPA (°)	18.0 ± 7.8	22.3 ± 4.7	0.083
SVA (mm)	18.7 ± 37.2	41.3 ± 32.2	0.097
Cobb angle (°)	15.0 ± 8.2	10.4 ± 7.9	0.144
CBD (mm)	17.9 ± 13.4	18.0 ± 11.7	0.984
Postoperative–preoperative (Δ)			
Δ TK (°)	19.8 ± 8.8	19.6 ± 9.0	0.937
Δ LL (°)	41.3 ± 11.2	22.8 ± 11.3	** < 0.001**
Δ PT (°)	–17.0 ± 10.3	–7.1 ± 5.4	**0.003**
Δ PI (°)	–0.3 ± 2.8	0.6 ± 3.6	0.523
Δ PI–LL (°)	–38.1 ± 17.2	–21.6 ± 13.0	**0.008**
Δ TPA (°)	–22.5 ± 13.0	–10.3 ± 8.4	**0.006**
Δ SVA (mm)	–98.6 ± 60.8	–47.9 ± 54.5	**0.029**
Δ Cobb angle (°)	–25.9 ± 8.9	–13.1 ± 9.5	**0.001**
Δ CBD (mm)	–29.8 ± 39.9	–6.7 ± 15.8	**0.044**

Data are shown as mean ± SD. PAP, posterior-anterior–posterior; AP, anterior–posterior; TK, thoracic kyphosis; LL, lumbar lordosis; PT, pelvic tilt; PI, pelvic incidence; TPA, T1 pelvic angle; SVA, sagittal vertical axis; CBD, coronal balance distance. *P values were calculated using the unpaired t-test

### Patient-reported outcomes

The SRS-22 outcomes in the two groups are shown in Table 5. At baseline, the PAP group had significantly lower SRS-22 scores for self-image and mental health than the AP group. However, the SRS-22 scores became comparable in the two groups at the 2-year follow-up.

**Table 5:** Patient-reported outcome measures

Variable	Two-stage PAP surgery (*N* = 12)	Single-stage AP surgery (*N* = 16)	*P* value*
Preoperative			
SRS-22 function	2.6 ± 0.7	2.7 ± 0.5	0.679
Pain	2.6 ± 0.6	3.0 ± 0.7	0.09
Self-image	1.9 ± 0.8	2.6 ± 0.9	0.048
Mental health	2.0 ± 0.8	2.8 ± 0.8	0.032
Satisfaction	3.3 ± 0.9	3.6 ± 0.6	0.628
Total	2.6 ± 0.4	2.7 ± 0.4	0.432
Postoperative			
SRS-22 Function	3.9 ± 0.5	3.8 ± 0.6	0.619
Pain	4.5 ± 0.3	4.3 ± 0.7	0.303
Self-image	3.9 ± 0.5	3.9 ± 0.7	0.957
Mental health	4.1 ± 0.4	4.0 ± 0.7	0.508
Satisfaction	4.3 ± 0.5	4.2 ± 0.5	0.424
Total	4.2 ± 0.3	4.1 ± 0.5	0.495

Data are shown as mean ± SD. PAP, posterior-anterior–posterior; AP, anterior–posterior; SRS, Scoliosis Research Society. *P values were calculated using the unpaired t-test

### Case presentation

**Fig. 3:**
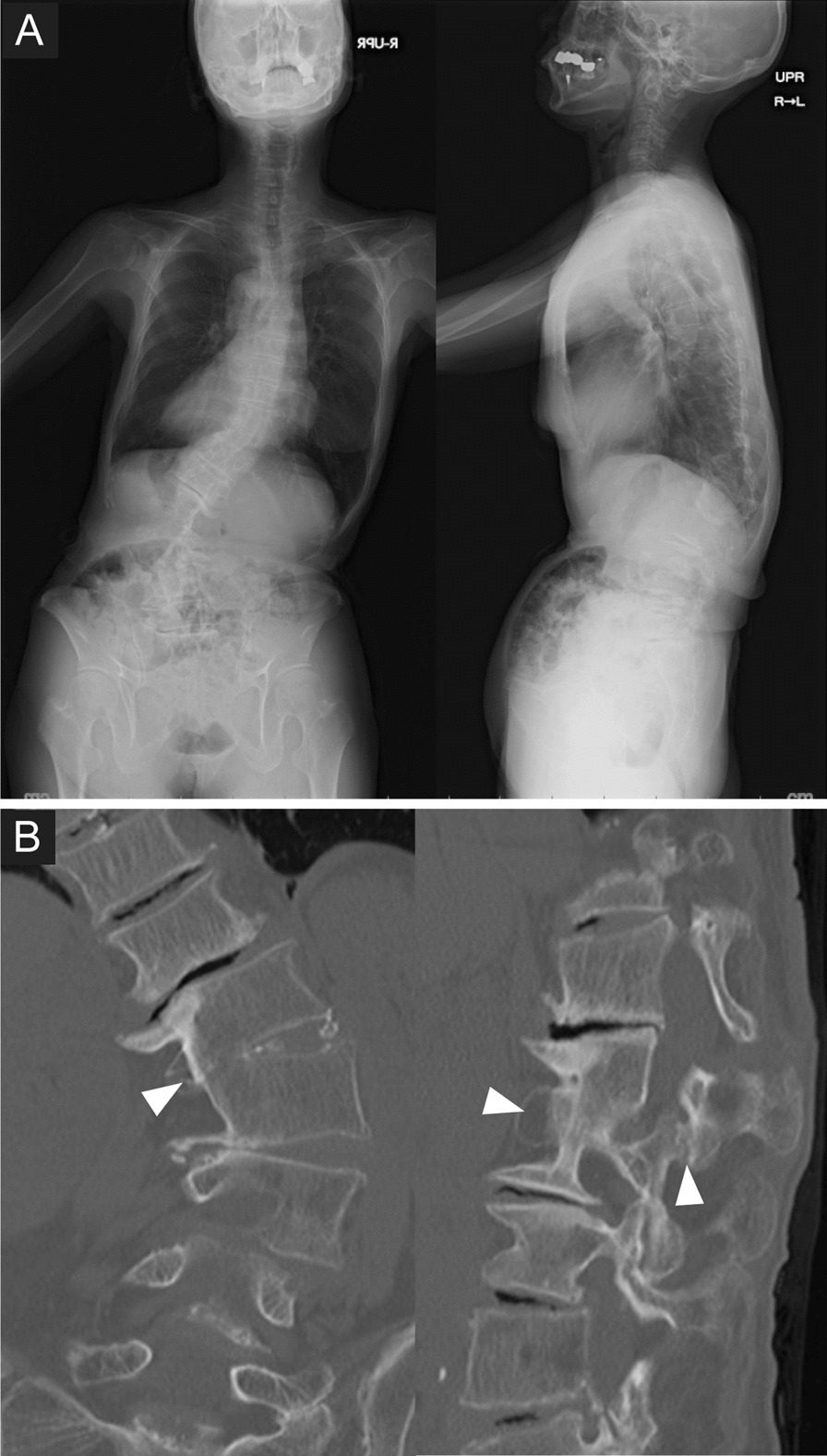
Preoperative images of a 73-year-old woman with rigid kyphoscoliosis treated by two-stage posterior-anterior–posterior surgery. **A** Preoperative free-standing posteroanterior and lateral spine radiographs. **B** Preoperative reconstructed CT images. Note that there is partial bony fusion of the L1/2 intervertebral disc and the facet joint (arrowheads)

**Fig. 4:**
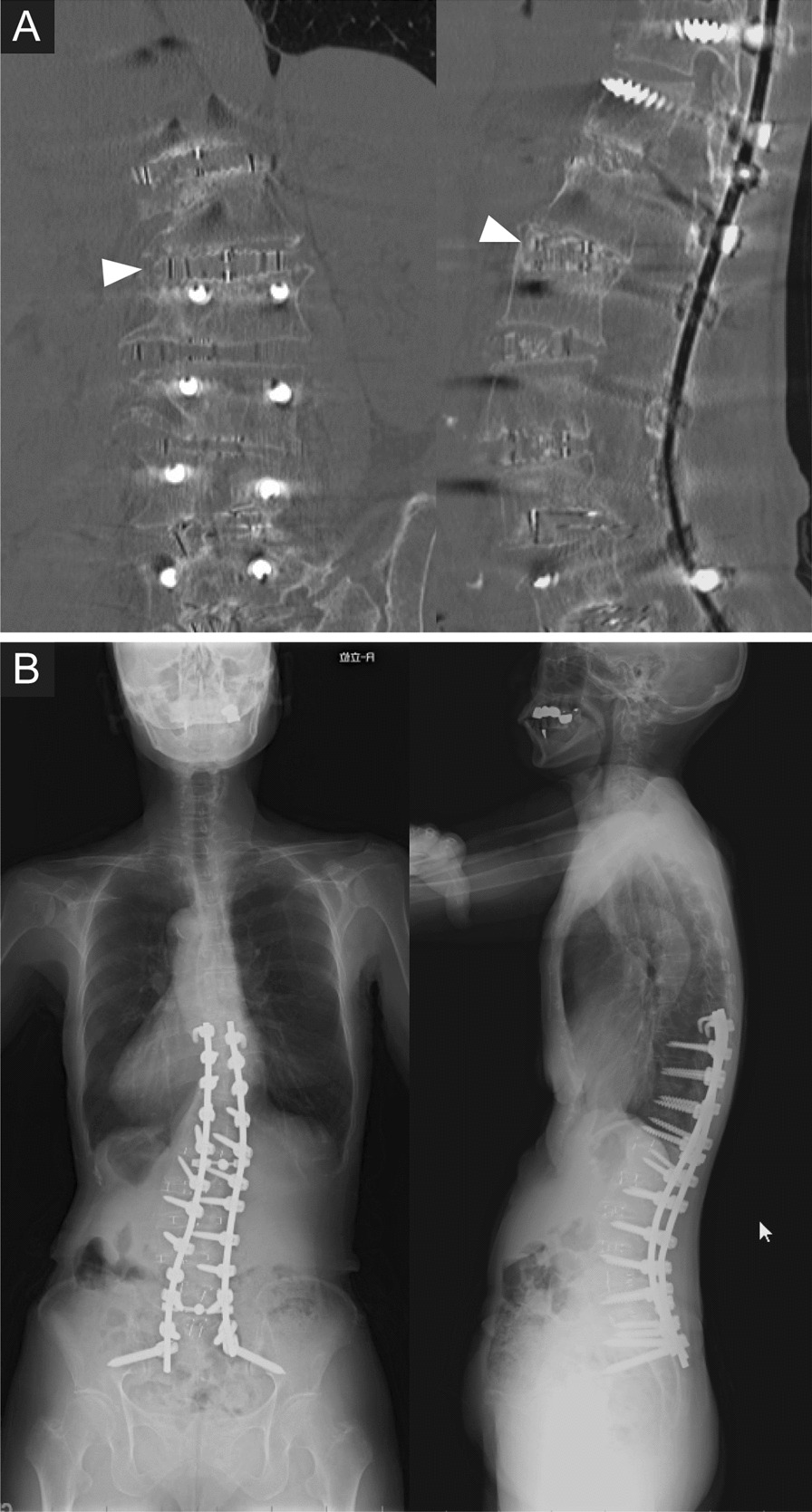
Postoperative images of a 73-year-old woman with rigid kyphoscoliosis treated by two-stage posterior-anterior–posterior surgery. **A** Postoperative CT demonstrates optimal placement of a lateral lumbar interbody fusion cage at the L1/2 level without endplate injury (arrowheads). **B** Free-standing posteroanterior and lateral spine radiographs obtained at the 2-year follow-up

We present a case of rigid kyphoscoliosis treated by two-stage PAP surgery. A 73-year-old woman was referred to our hospital for severe low back pain and gait disturbance. Preoperative radiography demonstrated degenerative lumbar kyphoscoliosis and sagittal malalignment (Fig. 2A). Preoperative CT demonstrated partial bony fusion of the L1/2 intervertebral segment (Fig. 2B). Postoperative CT showed optimal placement of the LLIF cage at the L1/2 level without endplate injury (Fig. 3A). At the 2-year follow-up, her preoperative symptoms had completely disappeared, and good global alignment was maintained (Figs. 3B, 4).

## Discussion

We investigated the effectiveness of two-stage PAP surgery using subcutaneously preserved autologous bone grafts compared with single-stage AP surgery. The key findings of this study were: (1) although PAP surgery was associated with a significantly longer operation time, the total EBL was significantly reduced in the PAP group compared with the AP group; (2) the rate of postoperative complications did not differ significantly between the two groups; (3) compared with the AP group, the PAP group showed significantly larger postoperative changes in radiological parameters, including LL, PT, PI–LL, TPA, SVA, Cobb angle, and CBD; and (4) compared with the AP group, changes in the sagittal Cobb angles in the PAP group were significantly larger at all lumbar spinal levels on CT-MPR images, suggesting that completing the posterior release prior to LLIF enhances the corrective power of LLIF for sagittal spinal deformities. Because under-correction of sagittal deformity is associated with worse health-related quality of life following ASD surgery [12], two-stage PAP surgery may be beneficial for patients with a large PI–LL mismatch.

Dividing one prolonged, complex surgery into two smaller procedures performed during one hospitalization is an intuitive strategy to reduce the risks of complications associated with protracted same-day surgery [13]. However, there are mixed results regarding the efficacy and safety of staged spine surgery for ASD. Consistent with our results, Rhee et al. showed that staged posterior surgery for complex deformity can be performed safely with little blood loss, few surgical complications, no major medical complications, and excellent outcomes [14]. In contrast, Spivak et al. reported that two-stage AP surgery for spinal deformity was associated with longer chest tube duration, increased anesthesia time, and increased hospital stay compared with single-stage surgery [15]. Passias et al. also concluded that staging circumferential spine surgery during the same hospitalization offers no mortality benefit and may even cause increased morbidity, including venous thrombosis, based on the Nationwide Inpatient Sample database [16]. While these studies provide some evidence, it is difficult to draw a conclusion regarding the safety of two-stage surgery because of limited patient matching between the two treatment groups. Surgeons are more likely to select two-stage surgery if a patient has complex deformity and multiple comorbidities.

Despite the mixed results regarding the complication rate, there are several reasons to justify the use of two-stage surgery for severe ASD. First, reducing the operative time on 1 day may mitigate the risk of coagulopathy due to hemodilution and hypothermia [17]. The significantly reduced total EBL in the two-stage PAP group may be attributed to the suppression of coagulopathy resulting from protracted same-day surgery. Second, dividing one prolonged procedure reduces the surgeon’s fatigue and improves performance during critical procedures, ultimately increasing the surgical safety [14]. Third, the increased magnitude and complexity of ASD surgery sometimes makes it challenging to accomplish a target correction within regular working hours in a single-stage manner. Fourth, staged surgery allows modifications of a surgical strategy based on the findings of the first surgery. For example, if a patient was prone to bleeding during the first surgery, surgeons and anesthesiologists can make sufficient preparations for the second surgery. Moreover, two-stage PAP surgery allows the correction of malpositioned screws in the secondary surgery. The avoidance of unplanned revision surgery is beneficial for patients.

In the present study, two-stage PAP surgery achieved significantly better deformity correction in the sagittal plane compared with single-stage AP surgery. The increased efficacy of deformity correction in PAP surgery is attributable to the completion of posterior release prior to LLIF cage insertion. Severe spinal deformity is commonly associated with hypertrophic degeneration of the facet joints [18]. Less mobile intervertebral discs due to hypertrophic facet joints may interfere with opening of the disc space and increase the risk of intraoperative endplate injury [19]. Sufficient posterior release prior to LLIF is particularly important if a patient has spontaneous fusion of kyphotic segments, as shown in the case presentation. In such cases, the typical AP procedure may involve an increased risk of endplate injury during LLIF cage insertion. Furthermore, posterior release prior to LLIF may be beneficial for patients with severe osteoporosis because reduced bone mineral density is a significant predisposing factor for endplate injury during LLIF procedures [20].

Our two-stage PAP surgery avoids ICBG harvest, which is beneficial because ICBG harvest potentially leads to numerous complications, including harvest-site morbidity, increased blood loss, iliac bone fracture, and incisional hernia [21]. While standard multilevel LLIF requires a large bone graft volume, the amount of local bone grafts obtained in the first surgery was sufficient for the loading of multiple LLIF cages. Various bone graft substitutes, such as synthetic bone and allogenic bone, can be used as substitutes or extenders of ICBG [22]. However, the use of bone substitutes generates an additional cost. The use of subcutaneously preserved bone grafts may not only reduce the surgical invasiveness but may also improve the cost-effectiveness of treatment.

This study has several limitations. First, this was a single-center retrospective study with a small sample size. Therefore, our between-group comparisons of radiological parameters and complication rates may not be sufficiently powered to achieve statistical significance. In particular, the present results regarding complication rates should be interpreted with caution. Second, the limited patient matching between groups (different severity of deformity at baseline) and potential for selection bias of single-stage versus two-stage surgery limit the generalizability of the data. A future prospective study that matches the baseline deformity severity between the two-stage PAP and single-stage AP surgery groups is needed to more accurately assess the treatment effects of two-stage PAP surgery. Finally, the safety and efficacy of subcutaneously preserved local bones remain to be confirmed. While several prospective studies have demonstrated the safety and viability of subcutaneously preserved cranial bone in patients undergoing cranioplasty [23, 24], further study is required to determine the safety of subcutaneously preserved local bone grafts for LLIF.

## Conclusions

The present results suggest that two-stage PAP surgery provides powerful correction of rigid kyphoscoliosis without increasing blood loss and postoperative complication rates. This method may be useful for patients with rigid deformity associated with hypertrophic facet arthritis and/or a large PI–LL mismatch.

## Data Availability

The datasets used during the current study are available from the corresponding author upon reasonable request.

## Abbreviations

ASD: Adult spinal deformity
LLIF: Lateral lumbar interbody fusion
PAP: Posterior-anterior–posterior
AP: Anterior–posterior
AI: Alar-iliac
PEEK: Poly-ether-ether-ketone
ASA-PS: American Society of Anesthesiologists physical status
ICBG: Iliac crest bone graft
UIV: Upper instrumented vertebra
EBL: Estimated blood loss
CT-MPR: Computed tomography multiplanar reconstruction
PJK: Proximal junctional kyphosis
SRS-22: Scoliosis Research Society–22
LL: Lumbar lordosis
SVA: Sagittal vertical axis
PI: Pelvic incidence
PT: Pelvic tilt
TPA: T1 pelvic angle
CBD: Coronal balance distance
